# Interploidy gene flow involving the sexual-asexual cycle facilitates the diversification of gynogenetic triploid *Carassius* fish

**DOI:** 10.1038/s41598-021-01754-w

**Published:** 2021-11-18

**Authors:** Tappei Mishina, Hirohiko Takeshima, Mikumi Takada, Kei’ichiro Iguchi, Chunguang Zhang, Yahui Zhao, Ryouka Kawahara-Miki, Yasuyuki Hashiguchi, Ryoichi Tabata, Takeshi Sasaki, Mutsumi Nishida, Katsutoshi Watanabe

**Affiliations:** 1 Laboratory of Animal Ecology, Graduate School of Science, Kyoto University, Sakyo-ku, Kyoto, 606-8502 Japan; 2 Research Institute for Humanity and Nature, Kita-ku, Kyoto, 603-8047 Japan; 3 Department of Marine Biology, Tokai University, Shimizu, Shizuoka, 424-8610 Japan; 4 Atmosphere and Ocean Research Institute, University of Tokyo, Kashiwa, Chiba 277-8564 Japan; 5 Graduate School of Fisheries and Environmental Sciences, Nagasaki University, Nagasaki, 852-8521 Japan; 6 Key Laboratory of Zoological Systematics and Evolution, Institute of Zoology, Chinese Academy of Sciences, Chaoyang District, Beijing, 100101 China; 7 NODAI Genome Research Center, Tokyo University of Agriculture, Setagaya-ku, Tokyo, 156-8502 Japan; 8 Department of Biology, Osaka Medical and Pharmaceutical University, Takatsuki, Osaka 569-0801 Japan; 9 Graduate School of Human and Animal-Plant Relationships, Tokyo University of Agriculture, Atsugi, Kanagawa 243-0034 Japan; 10 University of the Ryukyus, Nakagami-gun, Okinawa, 903-0213 Japan; 11 Present Address: Laboratory for Chromosome Segregation, RIKEN Center for Biosystems Dynamics Research, Chuo-ku, Kobe, 650-0047 Japan; 12 Present Address: Lake Biwa Museum, 1091 Oroshimo, Kusatsu, Shiga 525-0001 Japan

**Keywords:** Population genetics, Biogeography, Evolutionary ecology

## Abstract

Asexual vertebrates are rare and at risk of extinction due to their restricted adaptability through the loss of genetic recombination. We explore the mechanisms behind the generation and maintenance of genetic diversity in triploid asexual (gynogenetic) *Carassius auratus* fish, which is widespread in East Asian fresh waters and exhibits one of the most extensive distribution among asexual vertebrates despite its dependence on host sperm. Our analyses of genetic composition using dozens of genetic markers and genome-wide transcriptome sequencing uncover admixed genetic composition of Japanese asexual triploid *Carassius* consisting of both the diverged Japanese and Eurasian alleles, suggesting the involvement of Eurasian lineages in its origin. However, coexisting sexual diploid relatives and asexual triploids in Japan show regional genetic similarity in both mitochondrial and nuclear markers. These results are attributed to a unique unidirectional gene flow from diploids to sympatric triploids, with the involvement of occasional sexual reproduction. Additionally, the asexual triploid shows a weaker population structure than the sexual diploid, and multiple triploid lineages coexist in most Japanese rivers. The generated diversity via repeated interploidy gene flow as well as an increased establishment of immigrants is assumed to offset the cost of asexual reproduction and might contribute to the successful broad distribution of this asexual vertebrate.

## Introduction

The ubiquity of sexual reproduction in the eukaryotes is one of the great puzzles in evolutionary biology because the costs of sexual reproduction (the cost of producing males and investing time and energy in finding a mate) are immediate, whereas its potential benefits (facilitation of adaptation to changing environments, elimination of deleterious mutations) are delayed^1–3^. A large body of literature aimed at explaining the maintenance of sex has generally treated sexual and asexual reproduction as mutually exclusive strategies and has predicted that asexuality is an evolutionary dead end^4,5^. However, recent findings regarding long-surviving asexual lineages and their genetic diversity have changed the view of the evolutionary potential of asexual taxa^6^. The genetic diversity in these long-surviving asexual species is facilitated by rare sexual reproduction^7,8^; transmission of genetic elements from related sexual taxa (e.g., paternal introgression)^9–12^; or in some cases, mitotic recombination^13^. Exploring the mechanisms underlying the generation and maintenance of genetic diversity in asexual organisms is essential for understanding the ecological and evolutionary relevance of the alternative reproductive modalities, leading to the identification of the proximate forces shaping the evolution of sex.

Asexual vertebrates are of particular interest to biologists due to both a fascination with their rarity and anticipation that such exceptions deepen our understanding of sexual reproduction^3,14–17^. One type of asexual reproduction found in vertebrates is gynogenesis, in which offspring are formed parthenogenetically, yet egg development cannot be completed without sperm from a related bisexual species that degenerates without fusing with the egg nucleus^3^. Sperm-dependent asexual species can only persist in sympatry with a closely related sexual host that offers a continuous supply of males. Thus, gynogenesis suffers from certain disadvantages of sexuality (e.g., finding mates, sexual diseases) along with those of asexuality, which should make it difficult for these lineages to show long-term persistence^18^.

The gynogenetic triploid found in the genus *Carassius* (Teleostei: Cyprinidae) is a good model for studying the evolution of asexual vertebrates. This genus is taxonomically confusing, but at least three species are recognized: the wild goldfish *Carassius auratus*-complex (sometimes referred as *C. gibelio, C. langsdorfii*, and *C. buergeri*^19^), the Japanese white crucian carp (*C. cuvieri*), and the crucian carp (*C. carassius*). Among them, much attention has been focused on the *C. auratus-*complex exhibiting sexual and gynogenetic reproduction associated with ploidy polymorphism (sexual diploid, gynogenetic triploid, and rare tetraploid)^20,21^. This gynogenetic triploid is one of the most widespread asexual vertebrates, which is distributed in and around Eurasia, including Taiwan and the Japanese islands, and uses sympatric sexual diploid *Carassius* fish as sperm donors^20,22^. Substantial morphological and genetic variability has been reported in the gynogenetic triploid *Carassius*^23–25^. Intriguingly, phylogenetic studies on mitochondrial DNA reported that sexual diploids and gynogenetic triploids shared various mitochondrial haplotypes over diverged clades in wide ranges of East Asian freshwaters^26,27^. These results indicate underlying mechanisms that facilitate genetic diversity or multiple autotriploidization-like origins from sexual diploid populations despite the extreme rarity of asexual vertebrates^3^.

**Figure 1:**
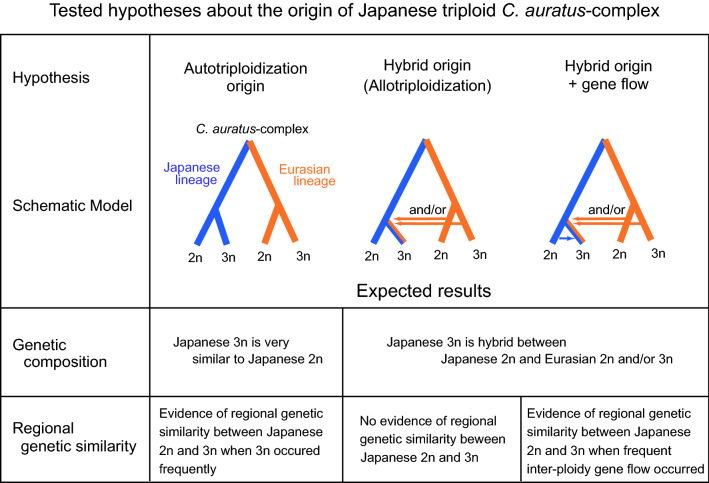
Tested hypotheses about the origin of the Japanese triploid *C. auratus*-complex investigated in this study and expected analysis outcomes.

The objective of this research was to elucidate how the gynogenetic triploid *Carassius* fish has spread widely and avoided extinction by examining the origin of this fish in the Japanese archipelago and the mechanisms facilitating its genetic diversity. Integrating the dataset of nuclear loci obtained by target resequencing and transcriptomes, mitochondrial sequences, microsatellites and morphological measurements generated from geographically comprehensive specimens (Supplementary Fig. S1, Table S1), we address two main questions: (i) did gynogenetic triploid *Carassius* originate from hybridization? Although nearly all asexual vertebrates are of hybrid origin^15^, there are two hypothetical scenarios for the origin of the Japanese gynogenetic triploid *Carassius*—one is the hybrid origin implied by the different genetic characteristics between Japanese diploids and gynogenetic triploids (e.g., allozyme^21^; RAPD^28^), and the other is autotriploidization, i.e., the occurrence of triploid individuals from a single diploid population^27,29^. We examined the genetic composition of the Japanese gynogenetic triploid to test for presumable admixtures between Japanese and Eurasian lineages (Fig. 1). (ii) What mechanisms facilitate the genetic variability in triploid *Carassius*? One possible cause is the multiple origins of gynogenetic triploids via autotriploidization, which will result in superficially elevated genetic diversity among independently originated triploids. Another potential non-mutually exclusive mechanism for generating genetic diversity among triploid *Carassius* is the transmission of genetic elements (gene flow), including mitochondrial DNA, from related sympatric sexual species, as suggested for several invertebrates^8^ and plants^7,30^. Under the autopolyploidization scenario, sympatric pairs of diploid and triploid are expected to have high genetic similarity and they should be almost indistinguishable. Conversely, in the case of hybrid origin with gene flow, although the sympatric pairs of diploid and triploid are also expected to have some geographical genetic cohesion, they would be distinguished by traces of ancestral parent alleles in triploids (Fig. 1). Thus, we examined the regional genetic similarity of diploid and triploid *C. auratus*-complex collected mainly from Japan by using nuclear microsatellite DNA and mitochondrial DNA haplotype frequency data. Specifically, we tested the presence of isolation-by-distance (IBD) between diploid and triploid *Carassius*. We further evaluated the frequency of coexistence of genetically distinct triploids and clarified the population structure of *Carassius* fishes to evaluate the contributions of selection and migration in the maintenance of the diversity as possible key factors enabling triploid *Carassius* to thrive.

## Results

### Genetic composition of gynogenetic triploid *Carassius*

**Figure 2:**
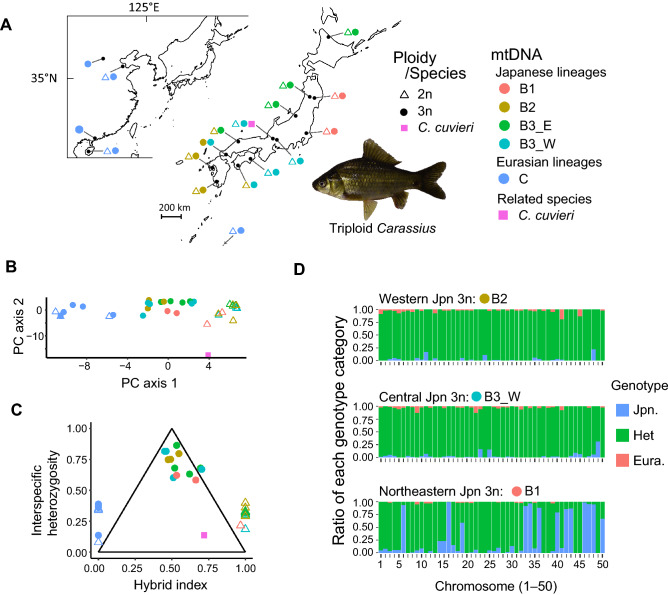
Genetic composition of triploid *Carassius*. (**A**) Locality, ploidy, and mitochondrial lineages of specimens used for target resequencing. Triangles indicate diploids, circles represent triploids, and a square represents *C. cuvieri* with colors corresponding to the mitochondrial lineages of the specimens. The map was generated using the R package mapdata. (**B**) PCA plot and (**C**) triangle plots of interspecific heterozygosity versus hybrid index based on the SNPs obtained from targeted resequencing. (**D**) Genetic composition across chromosomes in selected specimens of major mtDNA lineages of triploid *Carassius* in Japan based on dSNPs generated from RNA-seq. The ratios of genotype categories from dSNPs between the Japanese and Eurasian lineages are colored as Japanese homozygote (blue), Eurasian homozygote (red), and heterozygote (green).

To characterize the genetic composition of gynogenetic triploid *Carassius*, we conducted target resequencing on available nuclear markers used in Cyprinidae phylogeny^31^ and previous studies on *Carassius*^27,32,33^. The PC1 axis of principal component analysis (PCA) based on 1,100 informative single nucleotide polymorphisms (SNPs) from 30 nuclear markers of 15 sexual diploid and 19 gynogenetic triploid *Carassius* (Fig. 2A) represented the difference between Japanese diploid and Eurasian diploid/triploid lineages, and Japanese triploids appeared intermediate, and PC2 represented the difference between *C. auratus*-complex and *C. cuvieri* (Fig. 2B). Then, we further tested whether the intermediate PC1 values of Japanese triploids were due to the hybridization assuming Japanese diploids and Eurasian diploids/triploids as each parental lineage. There were 31 informative SNPs (*F*_*ST*_ > 0.75) spanning 14 markers between the assumed parental lineages (Supplementary Fig. S2). Strikingly, regardless of mitochondrial lineages of Japanese triploids (see below), it showed significant admixture with hybrid index values being 0.45 to 0.70 as well as high interspecific heterozygosity values (0.58–0.86) (Fig. 2C). These results indicated that Japanese triploids were hybrid between Japanese diploid and Eurasian diploid or triploid lineages, whereas no indication of hybridization was found in the Eurasian triploids.

Then, we further assessed the genome-wide distribution pattern of genotypes in Japanese triploids at diagnostic SNPs (dSNPs) between the putative parental lineages, Japanese diploids and Eurasian diploids/triploids. We used RNA-seq data conducted on six diploids and four triploids (see method; Supplementary Table S6) to retrieve genome-wide SNPs and found 2772 dSNPs on goldfish chromosome-level assembly; mean dSNPs per chromosome was 55.4 ranging from 7 to 128 (Supplementary Fig. S2). Consistent with the result of target resequencing, most genotypes of Japanese triploids at dSNPs were heterozygous especially in individuals collected from western and central Japan accounting for over 95% of dSNPs (western Japan: 96.3%; central Japan: 96.5%; northeastern Japan 74.3%; Fig. 2D). Interestingly, the individual collected from northeastern Japan showed the unique pattern in which the ratio of heterozygous genotypes greatly differed across chromosomes. That is, like the other individuals, the heterozygous genotype dominated in most of chromosomes (> 90% in 31 chromosomes), but a portion of chromosomes mainly consisted of the Japanese lineage genotype (50–90% in 5 chromosomes and > 90% in 8 chromosomes; Fig. 2D).

**Figure 3:**
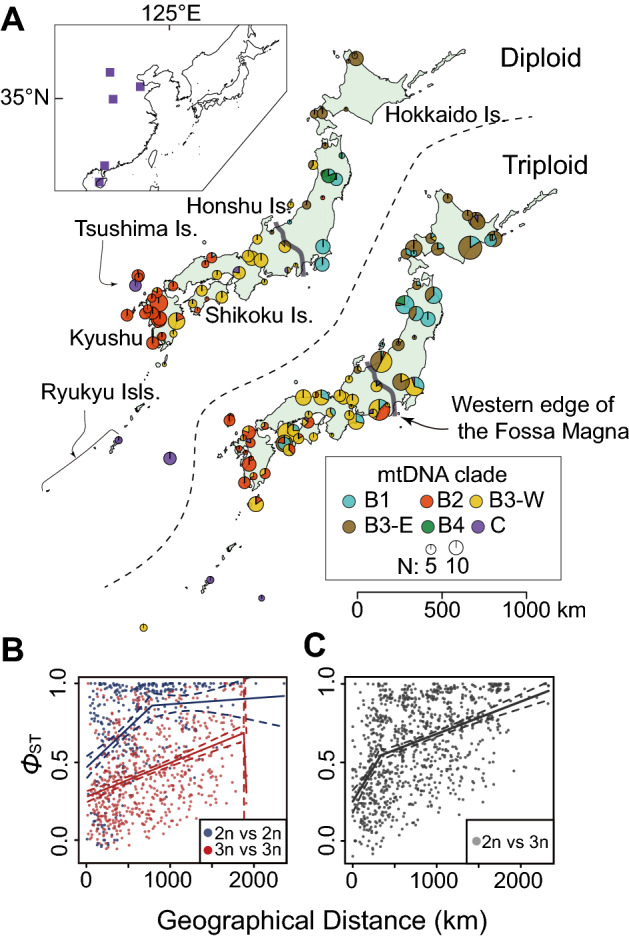
Population structure of *Carassius*, showing weaker geographical population structure of gynogenetic triploids than that of sexual conspecific diploids. (**A**) Geographic distribution of mitochondrial lineages of diploid and triploid *Carassius* in Japan. (**B**, **C**) Isolation by distance (IBD) in *Carassius* fish based on mtDNA haplotypes from 54 river systems in Japan. (**B**) IBD among diploids (blue) and triploids (red). (**C**) IBD between diploids and triploids. The maps were generated using the R package mapdata.

Genealogical analysis of mitochondrial genes analyzed on up to 933 *C. auratus*-complex specimens collected from 77 rivers in Japan and 4 rivers in Eurasia (Supplementary Table S1) yielded two previously reported superlineages within *C. auratus*-complex^26,34^: one distributed mainly on the Japanese main islands (Superlineage B in Supplementary Fig. S3) and the other on the Eurasian continent, Taiwan and the Ryukyus (Superlineage C). The Japanese superlineage included four divergent clades (Clades B1–B4), and diploid and triploid fish shared many haplotypes with geographical cohesion (Figs. 3, Supplementary S4). The mitochondrial Japanese lineages and the Eurasian lineages showed restricted sympatric zones (Fig. 3, Supplementary Table S1) despite the fact that almost all of the Japanese triploid specimens showed hybrid composition in the nuclear genome (Fig. 2). Such sympatric zones appeared in a few geographically sporadic rivers on mainland Japan and in the island at the boundary of the continental shelf (the Japanese lineage B2 and a Eurasian lineage on Tsushima Island; Figs. 3, Supplementary S3). The Eurasian mtDNA lineage on this island split from a Eurasian lineage, C1, around 1.11 (95% HPD, 0.58–1.82) Myr ago. Others found sporadically in mainland Japan had identical or very similar haplotypes as reported from the Eurasian continent (Supplementary Fig. S3), suggesting their origin by artificial introduction.

### Regional genetic similarity between diploids and triploids

Mitochondrial and nuclear isolation by distance (IBD) was inferred from the 933 *C. auratus*-complex specimens to test the genetic interaction between sympatric diploids and triploids. We estimated fixation index (*Ф*_*ST*_) values of mtDNA between sampling localities with discriminating ploidy, and IBD pattern was detected between and within diploid and triploid fish around Japan (Mantel test; diploid vs. triploid, *r* = 0.481, *p* < 0.0001; diploid, *r* = 0.420, *p* < 0.0001; triploid, *r* = 0.373, *p* < 0.0001; Fig. 3B, C). Triploids showed a weaker IBD pattern than that of diploids and presented a significantly lower pairwise *Ф*_*ST*_ value than diploids after controlling for the geographical distance by using a partial Mantel test (*r* = 0.108, *p* = 0.0016; Fig. 3B).

**Figure 4:**
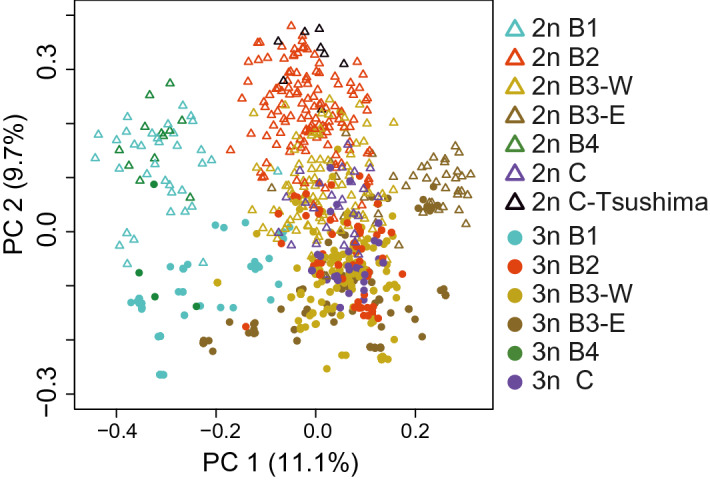
PCoA on mixed-ploidy populations of diploid and triploid *Carassiu*s using Bruvo’s distance performed in POLYSAT. Triangles indicate diploids, and circles represent triploids, with colors corresponding to the mitochondrial lineages of the specimens.

The genetic similarity among all specimens of diploid and triploid fish was examined based on 12 microsatellite genotypes. The genetic distance to the closest individuals indicates that 86.7% of 555 analyzed triploid specimens had genetically identical or very similar individuals (genetic distance < 0.1), as expected from their clonal reproduction, supporting robustness of our ploidy identification^23^ (Supplementary Fig. S5). In contrast, no diploid pairs showed an identical genotype (only 2.5% of 348 analyzed diploid specimens showed genetic distance < 0.1), indicating that stable clonal reproduction is limited to triploids. The results of principal coordinate analysis (PCoA) suggested that diploid specimens were clustered corresponding to mitochondrial lineages (B1 + B4, B2, B3; Fig. 4). Triploid specimens showed a tendency for differentiation from diploids on the PC2 axis, though they were genetically close to sympatric diploids on the PC1 axis. The consistent pattern was also inferred from PCA of the morphological characteristics of diploid and triploid fish. There were at least two distinct morphs in the Japanese *C. auratus*-complex, both of which were composed of diploid and triploid fish (Supplementary Fig. S6).

### Population structure and coexistence of triploid clones

**Figure 5:**
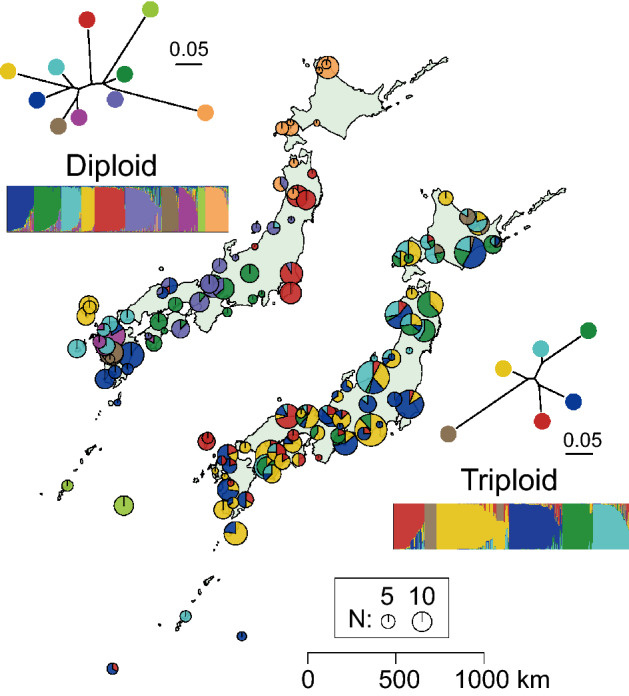
STRUCTURE results for diploid (*K* = 10, left) and triploid (*K* = 6, right) *Carassius* based on microsatellites. Relative sample sizes of clusters are shown as a pie chart on the map. NJ trees indicate allele frequency divergence between genetic groups inferred by STRUCTURE, and the tips are colored corresponding to the colors of clusters. In most of the river systems, triploid individuals are identified in multiple clusters. The maps were generated using the R package mapdata.

To infer population structure and estimate the frequency of coexistence of genetically distinct triploids, STRUCTURE analysis based on the 12 microsatellite loci was independently conducted for sexual diploid and gynogenetic triploid specimens collected from a wide area of Japan. Although this approach has a limitation in triploids due to the clonal inheritance, we expect that this analysis enables rough inferences about the genetic groups of genetically similar triploid lineages. Sexual diploids showed a distinct regional population structure (best selected *K* = 10), whereas a subtle regional structure with *K* = 6 was inferred for triploids (Fig. 5). In most of the river systems, triploid individuals from different genetic groups, including genetically diverged ones, were detected [triploid: 88% (36/41); diploid: 29% (9/31), only for diploid and triploid specimens with N ≥ 5; Fig. 5].

## Discussion

The present study investigated the genetic composition and historical origin of the gynogenetic triploid *Carassius* fish. Nuclear genetic analysis clearly revealed that Japanese gynogenetic triploid *Carassius* had a hybrid-like genetic composition between Japanese and Eurasian genetic groups (Figs. 2, Supplementary Fig. S2), consistent with several reports of different genetic characteristics (e.g., allozyme, RAPD) between diploids and gynogenetic triploids in Japan^21,28^. Thus, the autotriploidization scenario, i.e., independent multiple origins of triploids from diploids in each region, is not supported for the Japanese triploids. In contrast, for the triploids in Eurasia and the Ryukyu Islands, hybrid origin was not evidenced.

The hybrid-genome composition of Japanese triploids could be explained by recent artificial or historical natural hybridization scenarios. The explanation for the involvement of human activity assumes hybridization between local populations and artificially introduced individuals from the Eurasian continent. However, diploids identified as the Eurasian mtDNA lineages in mainland Japan were very rare, sporadic and not differentiated from the other Eurasian lineages. Thus, this effect cannot adequately account for the ubiquitous occurrence and genetic diversity of the triploids in Japan (this study and Refs.^21,26^). A more plausible cause is natural hybridization following secondary contact between the Japanese and Eurasian groups. Paleogeographic studies have suggested that a land bridge between mainland Asia and the Japanese islands emerged repeatedly during glacial periods of the Pleistocene^35,36^. We found the current coexistence of the Japanese and the unique Eurasian mtDNA lineage in the land bridge area (Tsushima Island; Figs. 3, Supplementary S3). This evidence supports the dispersal of the fish via the land bridge (e.g., river connections, reduced salinity in coastal areas, etc.). Such dispersal would have led to natural hybridization following secondary contact between Japanese diploids and Eurasian diploids and/or triploids and subsequent dispersal throughout the main Japanese islands.

This natural hybridization and dispersal scenario, however, does not fully explain the nuclear and mitochondrial genetic similarity with geographical cohesion between ploidies (Figs. 3, 4, Supplementary S4). If the gynogenetic lineage has not exchanged genes with diploid lineages, as is usually expected, such geographical cohesion between ploidies is unlikely. Thus, to adequately explain this genetic pattern, diploids and sympatric triploids must have genetic interaction in multiple regions. This interploidy gene flow should be basically unidirectional from diploid to triploid because no evidence of Eurasian genetic elements was detected in Japanese diploids.

In conclusion, the genetic variability among Japanese gynogenetic triploids was generated very likely through nuclear and mitochondrial gene flow from sympatric sexual diploid populations. Considering the hybrid genetic composition of the Japanese triploids and the interploidy gene flow, the primary origin of Japanese triploids is explained by the following two non-mutually exclusive scenarios: (i) colonized Eurasian triploids hybridized with Japanese diploids via interploidy gene flow, and/or (ii) triploidization by hybridization between Japanese and Eurasian diploids. Such events likely happened when a land bridge was formed during the glacial period. The gynogenetic lineages have persisted since it invaded the Japanese islands, where they dispersed throughout the archipelago and diversified.

The suggested interploidy gene flow is considered an important process for generating genetic diversity in triploids. The mtDNA haplotypes shared between sympatric ploidies are considered an introgression from diploids to triploids. This mtDNA introgression provides clues to revealing the unknown mechanisms underlying this directional gene flow. Due to the maternal inheritance of mtDNA, introgression of mitochondria from sexual diploids to gynogenetic triploids must satisfy two conditions: (i) the occurrence of males in the triploid lineage and (ii) reorigination of gynogenetic triploids from a cross between a male from the triploid lineage and a sexual diploid female. One plausible scenario is the involvement of tetraploid males. There is a low but significant proportion of mature tetraploids (0.44% of *Carassius* fishes in Lake Biwa^23^, and mature triploid and tetraploid males have been reported in natural habitats^37,38^). These tetraploids could result from the fertilization of unreduced eggs produced by a triploid female (3n) with haploid sperm (n) from a diploid male^39^. If this rare tetraploid male produces diploid sperm (2n), fertilization by such sperm of haploid eggs (n) from diploid females would lead to gynogenetic triploid individuals (Supplementary Fig. S7). This hypothesis is supported by the reported production of triploid offspring from crosses between a tetraploid male and a diploid female^38^, although the reproductive mode of these offspring has not yet been characterized.

Similar ploidy interconversion processes have been reported in a few invertebrates (planarian^8^), plants (dandelion^7^ and *Boehmeria*^30^), and vertebrates (the European cyprinid *Squalius alburnoides* complex^40,41^ and the North American cyprinid *Chrosomus eos-neogaeus* complex^42^). In triploid *Carassius*, the combination of interploidy gene flow and gynogenesis is assumed to make a large contribution to its genetic characteristics. The genome-wide F1-like hybrid genomic compositions of western and central Japanese *Carassius* triploids revealed by our RNA-seq are likely explained as a maintenance of the hybrid genome constitution via clonal inheritance or biased inheritance/selection in the process of interploidy gene flow. Moreover, other mechanisms such as recombination in the process of interploidy gene flow (e.g., when tetraploid males produce diploid sperm; Supplementary Fig. S7) are required to explain the loss of hybrid genome constitution in several chromosomes in the individual collected from northeastern Japan. To fully demonstrate the mechanisms of this interploidy gene flow and its consequences to their genome constitution of Japanese *Carassius*, further confirmation of the fertility and reproductive mode of the triploid offspring between a tetraploid male and a diploid female, as well as investigation of genetic composition based on extensive genome-wide analysis, are necessary.

The occurrence of multiple gynogenetic triploid lineages observed in most of the rivers (Fig. 5) is counterintuitive given the competitive exclusion principle, which predicts difficulty in the coexistence of closely related taxa that use a very similar niche^43^. Several studies on other asexual organisms have noted that environmental heterogeneity favors various clones with ecological differences (frozen niche variation model^44^), and the following negative frequency-dependent selection^45^ can be attributed to the maintenance of clonal lineage diversity in asexual organisms. These types of mechanisms, e.g., mechanisms associated with food habits^46^ and resistance to infection^12,47^, may facilitate diversity and the coexistence of multiple gynogenetic triploid lineages of *Carassius*. The genetic diversity in triploids could be reinforced by their enhanced connectivity of populations resulting from the increased establishment of immigrants after dispersal. For gynogenetic triploids, rare immigrants from geographically isolated populations maintain genetic independence from individuals of a recipient population, and the immigrants could be advantageous due to the anticipated negative frequency-dependent selection. By contrast, for sexual populations, alleles from such immigrants, even selectively favorable alleles, are often eliminated through genetic drift and recombination. This explanation is supported by the nuclear and mitochondrial less structured populations of triploids than those of diploids in *Carassius* despite their presumed similarity in ecological niches and dispersal ability (Figs. 3, 5). The widely extended metapopulation of triploids would contribute to the prolongation of the fixation time of clonal lineages through drift^48^.

The results of the present study suggest the presence of unique interploidy gene flow from sexual diploid to triploid *Carassius*; the gynogenetic triploids appeared to be generated repeatedly from mating between males derived from the gynogenetic triploids and sexual female diploids. However, independent acquisition of stable gynogenetic reproduction after the re-emergence of triploids appears to be evolutionarily challenging considering that the commonly reported mechanisms of gynogenesis in Japanese triploid *Carassius* require modifications of multiple fundamental cellular processes: suppression of polar body extrusion in meiosis I^49,50^ and no breakdown of the sperm nuclear envelope after fertilization^51,52^. Thus, genetic components (e.g., genetic variants, unique genome structure, and particular genome combinations related to hybridization) inherited from once-established gynogenetic triploid likely contribute to the observed frequent occurrence of new triploid clones. This inheritability of gynogenetic phenotype in natural populations of vertebrates is, to our knowledge, the first such evidence. The genetic diversity of Japanese triploids, generated by this interploidy gene flow, is considered to be maintained by the increased establishment of immigrants and probable negative frequency-dependent selection. Regarding the other gynogenetic animals, there is accumulating evidence of elevated heterozygosity counteracting the genetic degeneration process of Muller’s ratchet^1,18,53^, such as occasional leakages of a portion of the paternal genetic element^8–10,12^, balancing selection^12,45,47^, and selection on usage of hybrid genome (e.g., allelic expression)^54^. Similarly, gynogenetic *Carassius* could receive some of the benefits of sexual reproduction, counteracting the accumulation of deleterious mutations, as well as those of asexual reproduction. Importantly, geographically cohesive genetic similarity between ploidies and the expression of Japanese alleles in triploids suggest that gynogenetic *Carassius* utilize acquired alleles from their sexual hosts. The gene pool of the sexual host could become a bypassed venue for gynogenetic triploids to obtain alleles related to local adaptation (e.g., immunological adaptation, thermal adaptation) and sexual selection (e.g., morphological characteristics, sex pheromones, reproductive behaviors). Hence, occasional sexual reproduction and the effective use of acquired alleles, as well as increased migration, are considered key to avoiding extinction due to the genetic degradation and successful proliferation of this asexual vertebrate.

## Materials and methods

### Sampling and ploidy determination

All procedures were conducted in accordance with the Guidelines for Proper Conduct of Animal Experiments^55^, ARRIVE guidelines^56^, and approved by the Animal Experimentation Committee of Kyoto University. A total of 933 *Carassius auratus*-complex were collected from 77 river systems in Japan and 4 river systems in China from 2007 to 2016 (Supplementary Fig. S1, Table S1). All the maps used in this study were generated using the R package mapdata^57^. Ethanol-fixed tissues were used for total genomic DNA extraction using the Wizard Genomic DNA Purification Kit (Promega). The ploidy of *C. auratus*-complex specimens was estimated using the amplified number of alleles from 12 microsatellite loci^23,33^. This method had previously achieved > 97% matching with the result of flow cytometry in three geographically distant Japanese populations^23^. Genotypes were scored against the size standard ROX 400HD or LIZ 500 on an ABI 3130xl sequencer (Applied Biosystems) using GeneMapper software 3.0 (Applied Biosystems). The primers used for PCR amplification and the microsatellite genotypes are given in Supplementary Tables S2, S3.

### Genetic composition of triploids based on target resequencing of nuclear markers

We first screened the available nuclear markers used in Cyprinidae phylogeny^31^ and previous studies on *Carassius*^27,32,33^. Target resequencing of 30 well-amplified loci was conducted for 15 diploid and 19 triploid geographically representative *C. auratus*-complex specimens, in addition to *C. cuvieri* and *Cyprinus carpio* (Fig. 2A, Supplementary Tables S4 and S5). Sequencing libraries were prepared using the NEBNext Quick DNA Library Prep Master Mix Set for 454 (New England Biolabs) and sequenced on the GS Junior Sequencing system (Roche). All sequence data were submitted to the International Nucleotide Sequence Database Collaboration (INSDC) database (Supplementary Table S5; accession nos. DRX111446–DRX111481).

Reads from target resequencing were mapped against the target marker sequences of *Carassius* (Supplementary Table S4) using Bwa-men^58^ with default parameters after removing chimeric reads with USEARCH v8.1^59^ and trimming low-quality bases using fastp v0.20.0^60^ with the settings “-w 16 -3 -q 15 -l 50.” SNPs were called using HaplotypeCaller implemented in GATK v4.1.2.0^61^, and then SNPs were selected and filtered out with “QUAL < 20, QD < 2.0, MQ < 40.0, MQRankSum < -12.5.” After removal of sites with > 10% missing data using vcftools v0.1.16^62^, 1,100 bi-allelic SNPs were retained. Probabilistic principal component analysis (PCA) was conducted using the R package pcaMethods^63^.

To test the hybrid genetic composition of Japanese triploids, we calculated a hybrid index by mapping against interspecific heterozygosity. The hybrid index^64^ and interspecific heterozygosity were estimated from 103 commonly observed SNPs (minor allele frequency > 0.3 in all 35 sequenced *Carassius* specimens) using est.h (fixed = FALSE) and calc.intersp.het functions, respectively, implemented in the R package Introgress^65^. The est.h function uses a maximum likelihood method to estimate the proportion of genome with alleles derived from one of the assumed ancestry populations, whereas the calc.intersp.het function estimate the proportion of genome with alleles inherited from both ancestry populations. For the analysis of hybrid genetic composition, 12 Japanese diploids and eight Eurasian diploids/triploids were used as potential admixed ancestries based on the result of PCA. *F*_*ST*_ values for each SNP between the putative ancestry lineages were calculated using vcftools.

### Genetic composition of triploids across chromosomes based on whole transcriptome

To characterize the genotype composition of Japanese triploids across chromosomes, we assessed their genotypes using diagnostic SNPs (dSNPs) between their putative parent lineages (i.e., Japanese diploids and Eurasian diploids/triploids). We used RNA-seq to obtain these genome-wide dSNPs. Liver total RNA was extracted from the pairs of diploid and triploid mature female *C. auratus*-complex specimens belonging to three major Japanese and one Eurasian mtDNA lineages^26,34^ using the RNeasy Lipid Tissue Mini Kit (Qiagen). These specimens were different from those used for the target resequencing, but they were collected from the same river systems except for the Ryukyu islands (Supplementary Table S6). All samples had an RNA integrity number (RIN) of 8.5 or better on the 2200 TapeStation (Agilent). RNA-seq libraries were constructed using the NEBNext Ultra RNA Library Prep Kit for Illumina (New England Biolabs), and were sequenced on an Illumina HiSeq 2500/4000 with 100-bp paired-ends. One of the Japanese diploid individuals was further sequenced on Miseq with 300-bp paired-end reads. All sequence data were submitted to the INSDC (Table S6; accession nos. DRX111437–DRX111445). Additionally, the deposited RNA-seq data of the goldfish, SRX668453^66^, and gibel carp, SRR922167^67^ were downloaded.

After removal of adapter and low-quality bases using fastp with the option “-3 -q 15 -l 50,” the RNA-seq reads from the diploid and triploid *C. auratus*-complex specimens were mapped to the goldfish genome^68^ using STAR v2.7.9a^69^. We follow the GATK Best Practices workflow for RNA-seq^70^ with slight modification. First, to facilitate mapping reads, a splice junction table was created by mapping all RNA-seq reads to the goldfish genome with the options “alignSJDBoverhangMin 1 and outFilterMismatchNmax 999.” Then, with the aid of the created splice junction database, we mapped the reads from each *Carassius* specimen with the parameters “alignSJoverhangMin 8, alignSJDBoverhangMin 1, and outFilterMismatchNmax 999.” After filtering out multi-mapping reads using samtools v1.10^71^, SNPs were called using HaplotypeCaller implemented in GATK with the option “–dont-use-soft-clipped-bases,” and then jointly genotyped. Next, SNPs were selected and filtered out with “FS > 30, QD < 2.0, -window 35, -cluster 3, DP > 20, GQ > 20,” resulting 66,451 bi-allelic SNPs. Then, dSNPs located on the goldfish chromosomes-level assembly were developed by calculating *F*_ST_ and exploring fixed alleles (*F*_ST_ = 1) between the putative parents (three lineages of Japanese diploids vs. four Eurasian diploids/triploids specimens) using vcftools. Genotypes of triploids from the resulting 2772 dSNPs were summarized for each chromosome and visualized using the R package ggplot2^72^.

### Mitochondrial gene sequence analysis

We sequenced partial gene regions of mitochondrial cytochrome *b* (cyt*b*) (885 bp) for all specimens. Representative samples of diploid and triploid *C. auratus*-complex specimens from the subgroups were further analyzed for the partial mitochondrial control region (CR) (297–316 bp) and the complete cyt*b* region (1141 bp). The primers used for PCR amplification and direct sequencing are given in Supplementary Table S7. All mitochondrial haplotype sequences have been deposited in the INSDC (Supplementary Table S8; accession nos.: cyt*b*, LC337605–LC337605; CR, LC337606–LC337660).

### Phylogenetic analysis of mtDNA

Genealogical reconstruction and estimation of the divergence time were conducted with mtDNA sequences of the CR and cyt*b* regions, with 218 operational taxonomic units (211 unique sequences of *C. auratus*-complex and 7 outgroup sequences), using BEAST version 1.8.4^73^. We imposed the minimum and maximum age constraints on the coalescence of the *Carassius* as 3.1–19.4 million years (Myr) ago in 95% interval (log-normal distribution prior, mean = 5, log SD = 1.1, and offset = 2.7) based on the fossil records of the *Carassius* (3.6–4.0 Myr ago^74^) and the estimated divergence time between *Carassius* and *Cyprinus* (11–20 Myr ago^68,75^). We adopted the strict clock model, giving the mean cyt*b* molecular clock rate of 0.76%/Myr/lineage (normal distribution prior, SD = 0.5%, ranging 0.3–1.3% in 95% interval) covering the range of cyt*b* clock rates in cyprinid fishes^76^. Further, we imposed the topological constraint of monophyly for *C. auratus*-complex^26^. The optimal substitution models for CR and cyt*b* were HKY + I + G and GTR + G + I, respectively, based on the Bayesian information criterion (BIC) in jModelTest v2.1.7^77^, and the Birth and Death model^78^ was adopted as the tree prior. The BEAST MCMC run was performed for 50 million generations, sampling every 2,000th generation with 25% discarded as burn-in. The consensus tree was calculated using TreeAnnotator v1.8.4 in the BEAST package.

### Population genetic analysis based on microsatellites

To test genetic interaction between diploid and triploid *C. auratus*-complex, regional genetic similarity between the ploidies were compared. The relationships among mitochondrial haplotypes of partial cyt*b* sequences were assessed by constructing a statistical parsimony network with a 95% connection limit using TCS version 1.21^79^. Fixation index (*Ф*_*ST*_) values between sampling localities with discriminating ploidy (only N ≥ 6 was used; diploid, 27 river systems; triploid, 37 river systems; Supplementary Table S9) were calculated by using ARLEQUIN ver. 3.5^80^. Mantel tests for the IBD trend in pairwise *Ф*_*ST*_ between populations were conducted by using the R package vegan with 10,000 permutations^81^. Differences in the IBD patterns between ploidies were investigated with partial Mantel tests^82^ by controlling for the geographical distance. Nonlinear changes in IBD patterns were analyzed with the broken-stick regression method using the R package segmented^83^.

Nuclear genetic similarity among diploid and triploid *Carassius* fishes was assessed based on genotypes from 12 microsatellite loci. The pairwise genetic distance between individuals was calculated using Bruvo’s distance^84^, as implemented in the R package POLYSAT^85^. This method considers the ambiguity of allele copy number in polyploids and works effectively with mixed-ploidy populations. The distance matrices obtained were used for principal coordinate analysis (PCoA). To further evaluate the frequency of coexistence of genetically distinct triploid lineages and clarify the population structure of *C. auratus*-complex, STRUCTURE analysis (v2.3.4)^86^ was carried out. Diploids and triploids populations were analyzed separately due to their difference in inheritance. For the triploid dataset, an ambiguity in allele copy number derived from a partial heterozygote was treated as two distinct alleles and a missing allele. Ten simulation runs were conducted for each of *K* = 3 to 20 using a length of burn-in of 10^6^ and 10^6^ replicates of MCMC. The optimum *K* was estimated by using the delta *K* method^87^ implemented in Structure Harvester^88^.

### Morphological analysis

As complementary evidence of gene flow, morphological characteristics were also compared among ploidies across regions using landmark-based geometric morphometrics. A total of 219 *C. auratus*-complex individuals collected from 11 localities that represented the population structure in Japan were used for the analysis. Eleven landmarks and the standard body length (SL) were recorded on each picture (c.f., Supplementary Fig. S6) with the tpsDig2 software^89^. Aligned Procrustes coordinates were used for generating a covariance matrix and were subjected to PCA using MorphoJ^90^.

## Supplementary Information

Supplementary Information 1.

Supplementary Information 2.

## Data Availability

All mitochondrial haplotype and nuclear gene sequences have been deposited in the INSDC (accession nos.: cyt*b*, LC337605–LC337605; CR, LC337606–LC337660). Target resequencing and RNA-seq data obtained in this study were also deposited to the INSDC (PRJDB6521). The datasets supporting this article are provided as part of the electronic supporting information.
