# Supplementary material for: Interploidy gene flow involving the sexual-asexual cycle facilitates the diversification of gynogenetic triploid Carassius fish
Source: Sci Rep. 2021 Nov 18;11:22485. doi: 10.1038/s41598-021-01754-w (PMC8602411; doi:10.1038/s41598-021-01754-w)
Supplement: Supplementary file 1 — Supplementary Information 1. [file 41598_2021_1754_MOESM1_ESM.pdf]

Figure S1

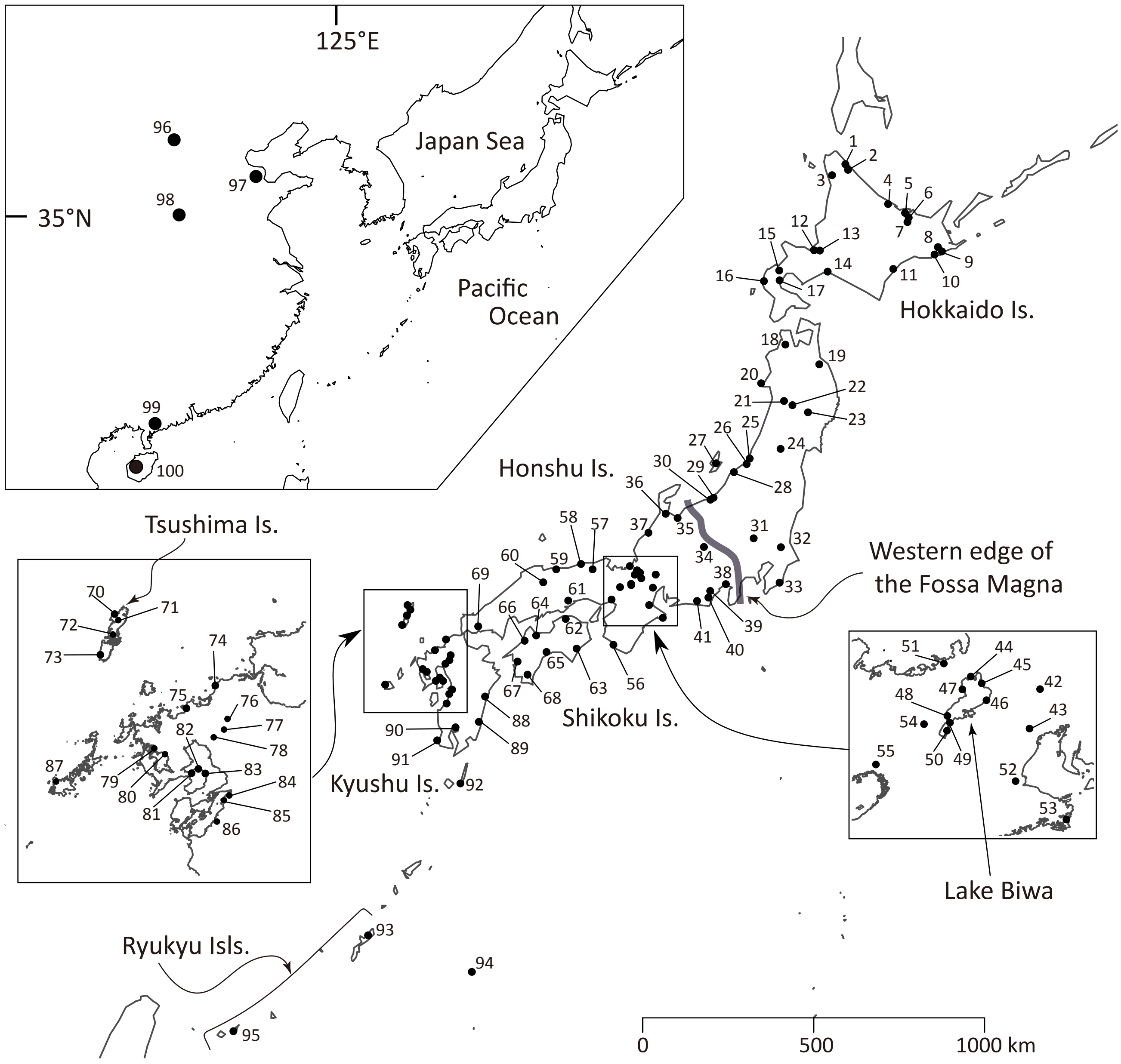

Figure S1. Sampling locations of *Carassius auratus*-complex, *C. cuvieri*, and *Cyprinus carpio*, with location codes (see Supplementary Table S1 for details).

Figure S2

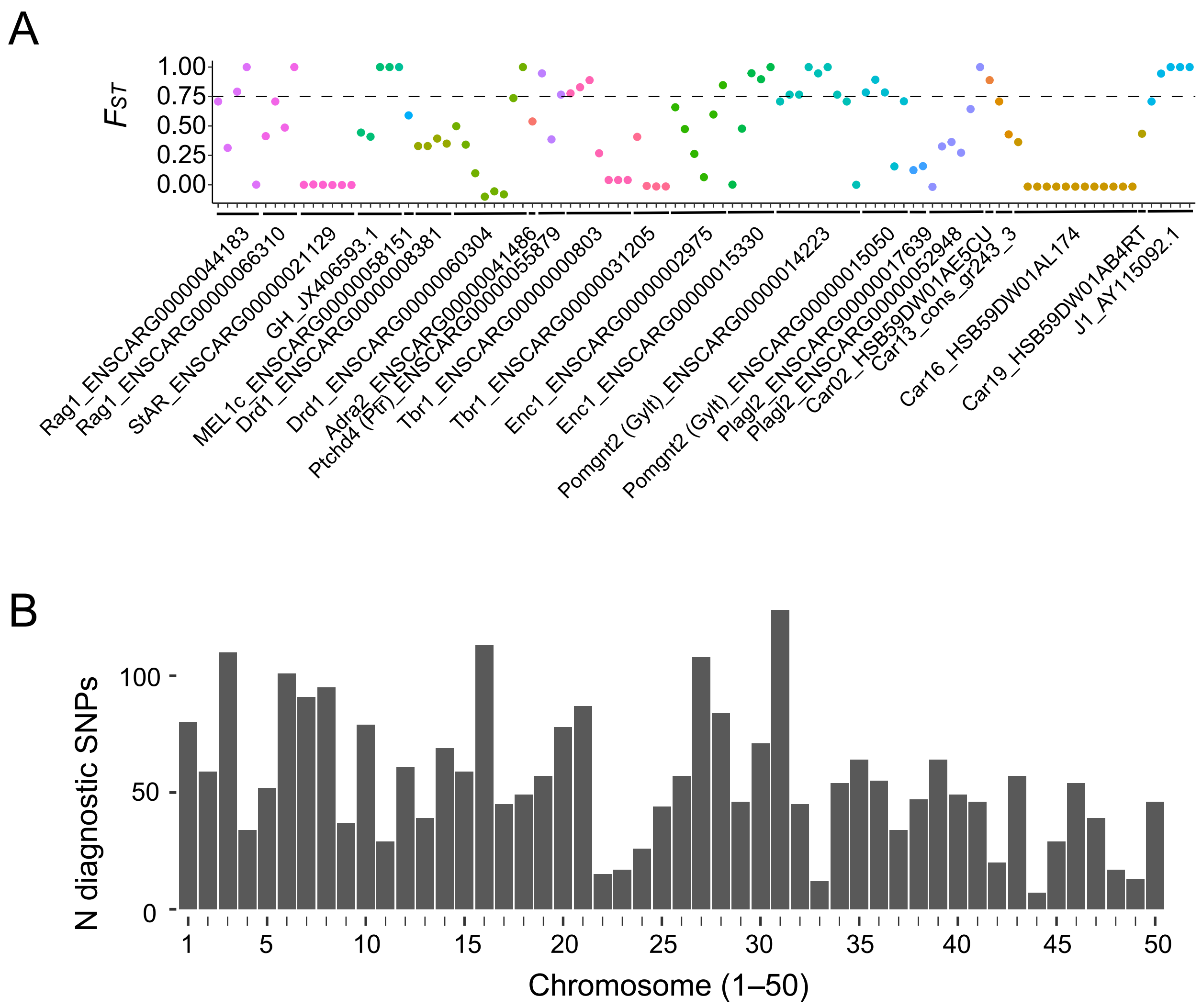

Figure S2. Distribution of diverged SNPs between Japanese diploids and Eurasian diploids/triploids lineages related Figure2. (A) F_{ST} across SNPs from target-resequencing. The dashed line indicates F_{ST} value of 0.75. Plots are colored according to their markers. (B) The number of diagnostic SNPs (F_{ST} = 1) per chromosome from RNA-seq data.

Figure S3-1

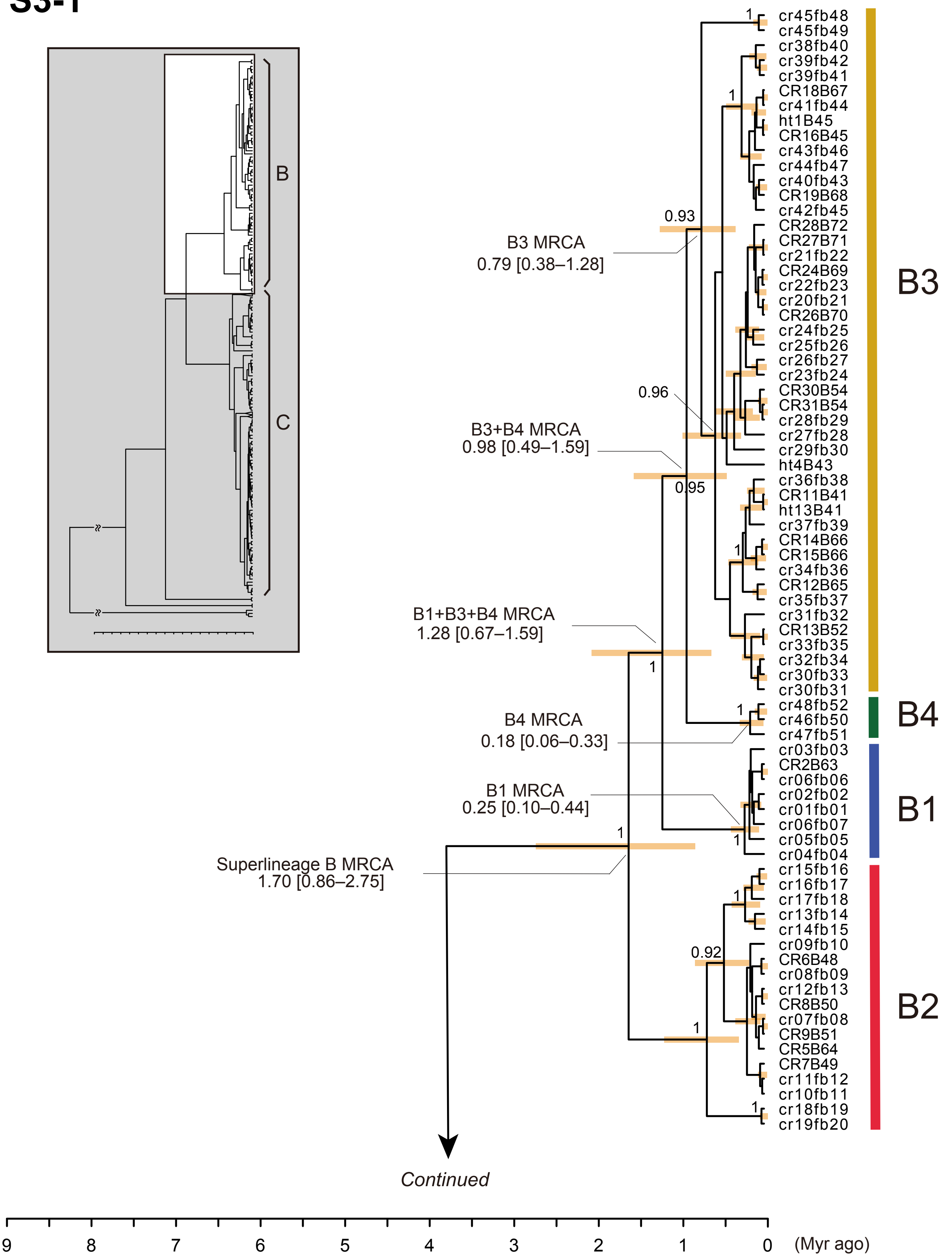

Figure S3.Phylogeny and divergence time of *Carassius* fishes around Japan resulting from a Bayesian strict clock analysis with mitochondrial DNA (complete cytochrome *b* + partial control region) sequences. Bars show 95% highest probability density (HPD) intervals of estimated age of most recent common ancestor (MRCA). Node supports are posterior probabilities of the Bayesian inference (shown when >85% for major nodes). Previously reported clades without any newly identified haplotypes were collapsed.

Figure S3-2

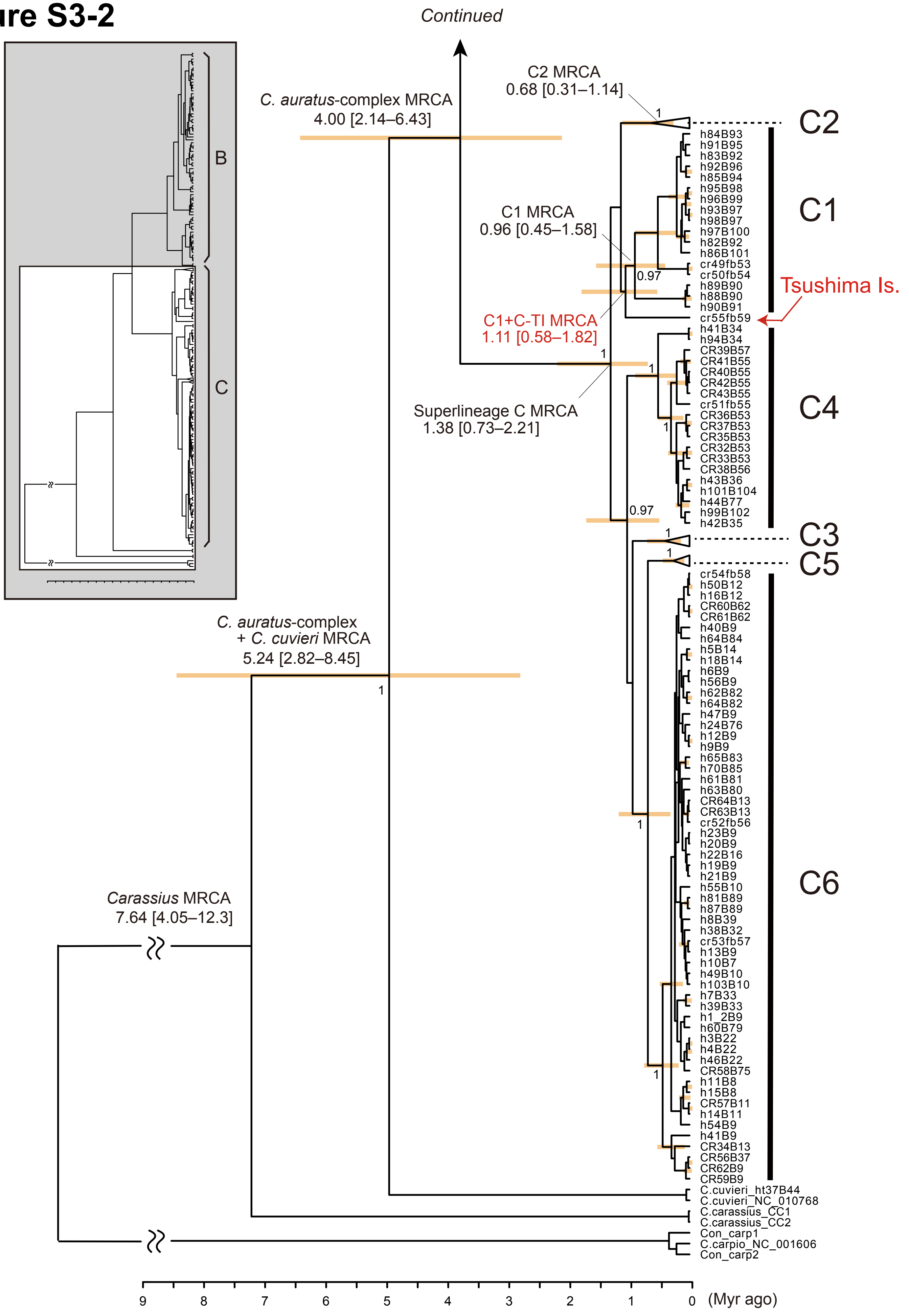

Figure S3. (continued)

Figure S4

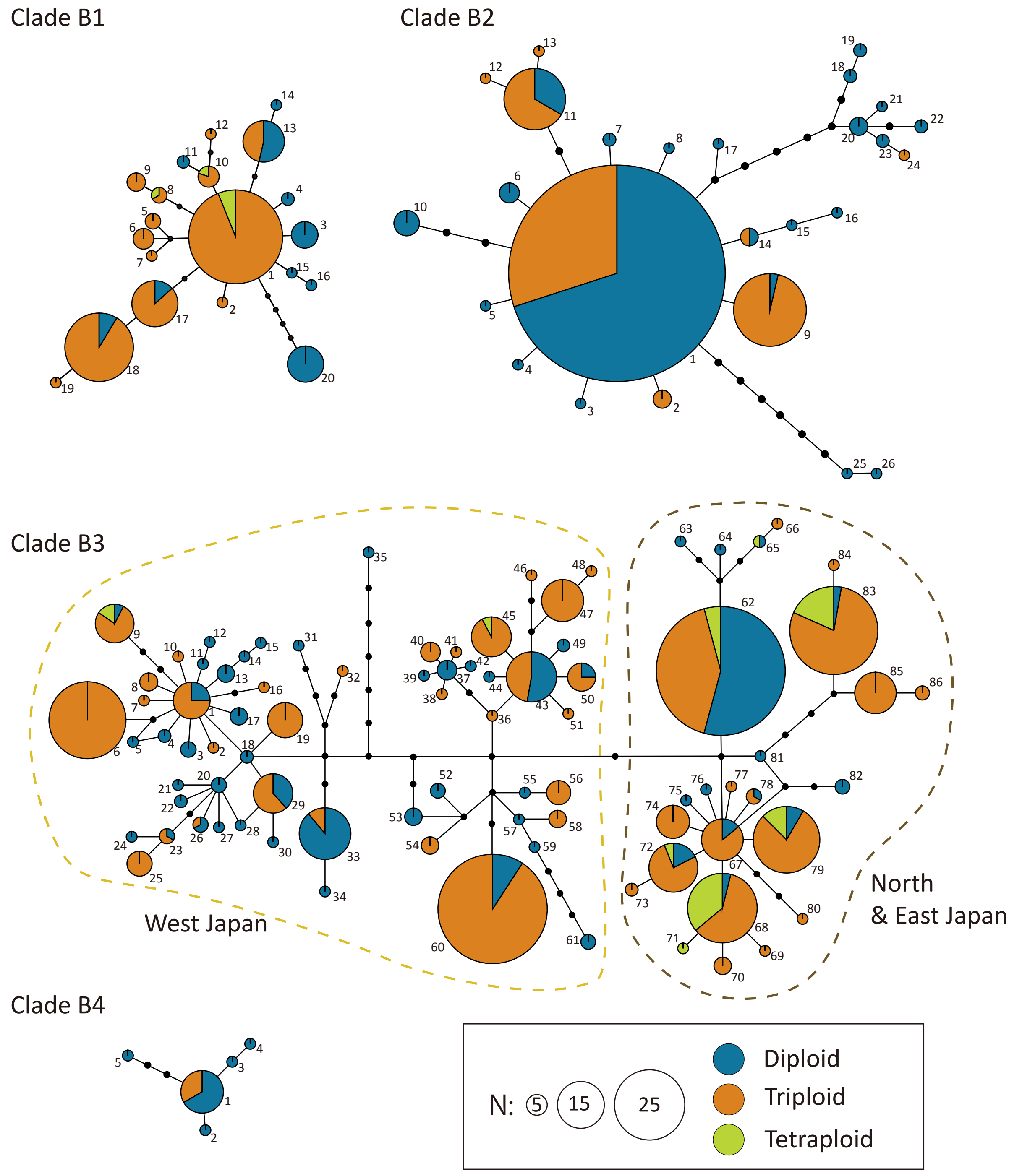

Figure S4. Mitochondrial haplotype networks for each lineage (Clade B1–B4) in mainland Japan showing diploid and triploid *Carassius* sharing diverse haplotypes. Each circle represents a unique haplotype, where size is proportional to sample size. Lines represent 1-bp differences, and black filled circles represent missing haplotypes not appearing in the samples. The relative sample sizes of diploid, triploid and tetraploid are shown as a pie chart, in which ploidies are distinguished by different colours. Codes of haplotypes, corresponding to those in Table S1, are shown.

Figure S5

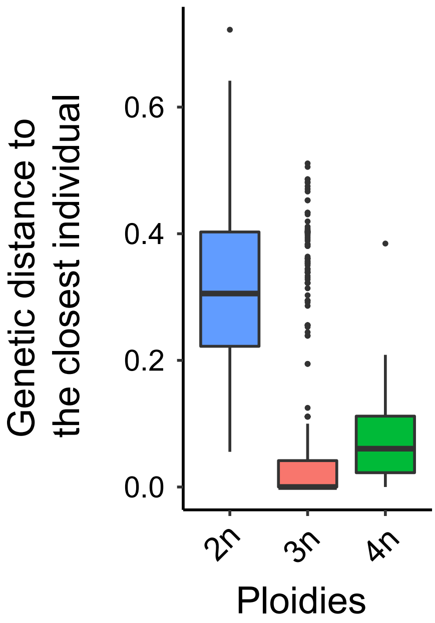

Figure S5. Genetic similarity between individuals for each ploidy of *Carassius* fish showing most of the triploids had an identical or very similar genotype to that of other triploid specimens.

Figure S6

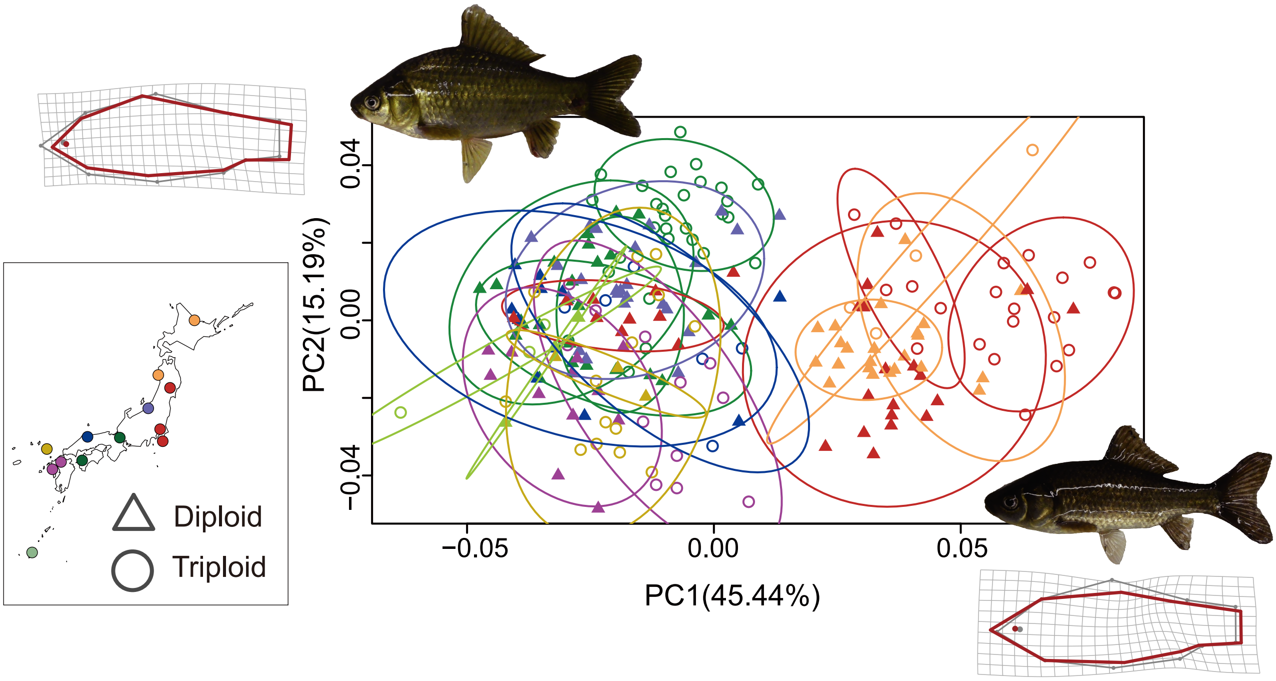

Figure S6. PCA of shape variation of *Carassius* fish showing similarity among diploid and triploid specimens across populations. Triangles indicate diploids and circles represent triploids with colored by corresponding genetic groups resulting from microsatellites of diploids (related to Fig. 5) with 90% confidence limit ellipses. Deformation plots for extreme points (red) and the mean (grey) of each axis are shown.

Figure S7

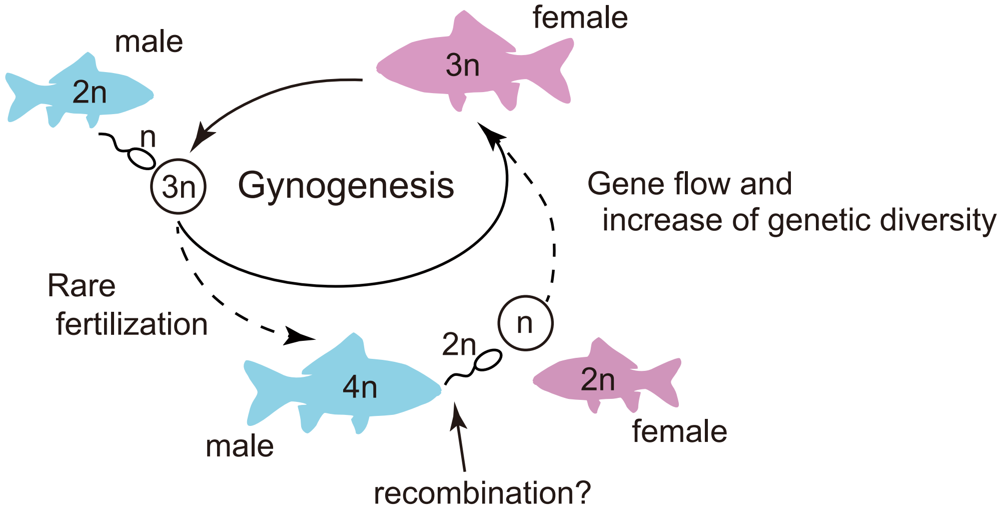

Figure S7. Expected interploidy gene flow from diploid to triploid *Carassius* fishe involving rare sexual reproduction
